# Neurologic Morbidity and Long‐Term Disability After Blunt Traumatic Aortic Injury: A Single‐Center Cohort Study

**DOI:** 10.1002/wjs.70302

**Published:** 2026-03-20

**Authors:** Lukas Mayer‐Suess, Maximilian Lutz, David Wippel, Kurt Moelgg, Florian Frank, Stefan Kiechl, Sabine Wipper, Elke R. Gizewski, Michael Knoflach, Martin Freund, Alexander Loizides, Florian Enzmann

**Affiliations:** ^1^ Department of Neurology Medical University Innsbruck Innsbruck Austria; ^2^ Department of Radiology Medical University Innsbruck Innsbruck Austria; ^3^ Department of Vascular Surgery Medical University Innsbruck Innsbruck Austria; ^4^ VASCage Research Center on Vascular Ageing and Stroke Innsbruck Austria

## Abstract

Blunt traumatic aortic injury (BTAI) is highly lethal, and contemporary management has shifted from open surgery toward thoracic endovascular aortic repair (TEVAR).

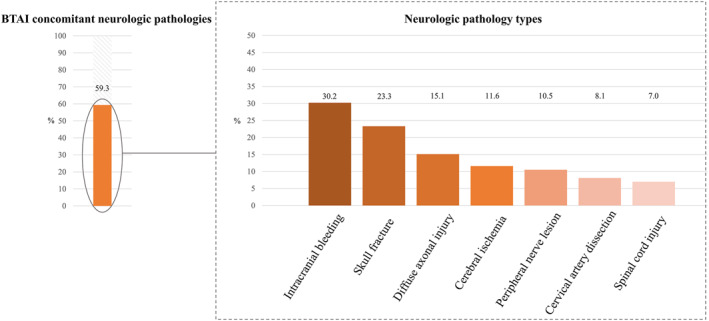

## 1 Introduction

Blunt traumatic aortic injury (BTAI) is highly lethal, and contemporary management has shifted from open surgery toward thoracic endovascular aortic repair (TEVAR) [1, 2]. Although aortic‐ and device‐related outcomes after TEVAR have been increasingly characterized, long‐term functional recovery after BTAI may be primarily driven by concomitant neurologic morbidity [3, 4, 5]. We therefore evaluated concomitant neurologic injury patterns and long‐term functional status in a consecutive BTAI cohort treated in the endovascular era.

## 2 Methods

We performed a retrospective cohort study of all patients treated for BTAI at the University Hospital of Innsbruck between 2005 and 2023. Potential cases were identified by keyword search (“TEVAR”, “thoracic stent graft/prosthesis”, “aortic rupture”) and included only if two independent raters confirmed BTAI. Demographics, trauma severity and treatment strategy were extracted from electronic records. CT imaging was used to assign BTAI Grades I–IV following the classification proposed by Azizzadeh et al. [6], consistent with recommendations in the Society for Vascular Surgery (SVS) guidelines [7]. Two board‐certified neurologists independently adjudicated neurologic diagnoses occurring concomitantly with blunt trauma and the respective outcomes, which were further categorized as traumatic brain injury (TBI) diagnoses (intracranial hemorrhage, diffuse axonal injury, and skull fracture) or potentially vascular‐ or treatment‐related cerebral ischemia (imaging‐confirmed acute ischemic stroke or transient ischemic attack). Further, occurrence of cervical artery dissection, spinal cord injury and peripheral nerve lesions (detected clinical and/or through electrophysiologic confirmation) was assessed. Where available, follow‐up of neurologic consequences was done. Herein, neurologic worsening during index hospitalization was defined as any increase in NIHSS or modified Rankin Scale (mRS) by ≥ 1 or a new neurologist adjudicated disabling neurologic deficit not captured by NIHSS or mRS values, both being widely used functional outcome measures in clinical neurology [8]. Long‐term functional outcome was assessed by mRS at last available follow‐up.

Lastly, the occurrence of TEVAR‐associated subclavian steal syndrome was recorded.

## 3 Results

Eighty‐six patients with CT‐confirmed BTAI were included (Table 1). The median age was 45.7 years (IQR 30.6–58.7) and 73/86 (84.9%) patients were male. Injury severity was substantial (Injury severity score [ISS] 24 [IQR 21–37]), whereas admission Glasgow coma scale (GCS) was benign overall (15 [IQR 9–15]). Most injuries were high grade (ESVS grade III/IV in 65/86, 75.6%). The predominant management strategy was TEVAR (72/86, 83.7%), with the remaining 14/86 (16.3%) patients being treated nSonoperatively. Median hospital length of stay was 15 days (IQR 8–28).

**TABLE 1:** Demographics and injury classification.

Variable	Overall (*N* = 86)
Age, years, median (IQR)	45.7 (30.6–58.7)
Male sex, *n* (%)	73 (84.9)
Injury severity score (ISS), median (IQR)	24 (21–37)
Glasgow coma scale (GCS) at admission, median (IQR)	15 (9–15)
BTAI grade (SVS), *n* (%)	
Grade I	5 (5.8)
Grade II	16 (18.6)
Grade III	62 (72.1)
Grade IV	3 (3.5)
TEVAR performed, *n* (%)	72 (83.7)
Nonoperative management, *n* (%)	14 (16.3)
Hospital length of stay, days, median (IQR)	15 (8–28)

Abbreviations: BTAI = blunt traumatic aortic injury, GCS = glasgow coma scale, IQR = interquartile range, ISS = injury severity score, SVS = society for vascular surgery, TEVAR = thoracic endovascular aortic repair.

**FIGURE 1:**
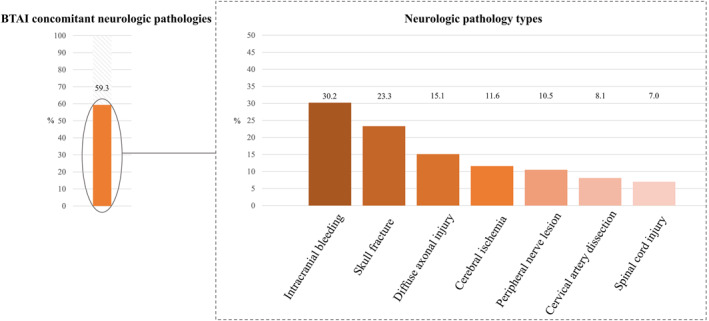
Overall frequency as well as type of concomitant neurologic injuries in patients with BTAI.

Concomitant neurologic injuries were identified in 51/86 (59.3%) patients (Figure 1). TBI diagnoses were most frequent: intracranial bleeding (26/86, 30.2%), diffuse axonal injury (13/86, 15.1%), and skull fractures (9/86, 10.5%). Mechanism‐of‐Injury‐related blunt cervical vascular injury was also common: 7/86 (8.1%) had cervical artery dissection, and in 2 cases it was defined as causal for subsequent cerebral ischemia. Separately, imaging‐confirmed cerebral ischemia occurred in 10/86 (11.6%) individuals, with five each being located in the anterior and posterior circulation, respectively.

Further concomitant injuries were peripheral nerve lesions, which occurred in 9/86 (10.5%) and spinal cord injury in 6/86 (7.0%). 5/6 (83.3%) of these were located at upper‐to mid‐thoracic levels (level T4–8) and one in cervical levels (C4). 4/6 (66.7%) spinal cord injuries were attributed to the initial trauma, whereas in two cases an association with TEVAR could not be excluded.

As it relates to neurologic follow‐up, 10/86 (11.6%) patients experienced neurologic worsening during index hospitalization, with intra‐hospital mortality being 8.1% (7/83). Among the different concomitant neurologic injuries, in‐hospital neurologic worsening was most frequent due to TBI‐pattern injury progression (6/10, 60%). In 42/79 (53.2%) hospital survivors, long‐term follow‐up was available with a median follow‐up duration of 6.8 years (IQR 4.3–10.0). No de‐novo or delayed neurologic deficits were identified during the follow‐up period. At last follow‐up, 19/42 (45.2%) reported persistent neurologic deficits with 17/19 (89.5%) relating to a functional limitation restricting daily activities (i.e. mRS > 1). When categorized by the affected system, residual deficits were central (CNS) in 10/42 (23.8%) and peripheral (PNS) in 9/42 (21.4%). Incomplete follow‐up was largely attributable to repatriation of nonresident patients.

Concerning treatment‐associated factors, grade III subclavian steal due to TEVAR‐related left subclavian artery coverage was documented in 33/72 (45.8%) individuals; only 3/33 (9.1%) were symptomatic at follow‐up, and symptoms were primarily consistent with upper‐extremity ischemia rather than posterior circulation events.

## 4 Discussion

In this consecutive BTAI cohort, concomitant neurologic injuries were common and largely reflected the high‐energy polytrauma mechanism: nearly 60% suffered neurologic injury, predominantly due to TBI‐pattern diagnoses or imaging‐confirmed cerebral ischemia. Importantly, the neurologic burden extended beyond hospitalization—as approximately 40% of patients with follow‐up had persistent disability limiting daily activities after a follow‐up of nearly 7 years. These findings highlight that BTAI is not solely an aortic emergency. BTAI can be considered as an indicator for a severe multisystem injury with frequently persistent neurologic consequences and primarily successful aortic repair does not necessarily translate into full recovery [4, 5, 9]. However, our data also suggest that neurologic burden carried by patients after BTAI relates to the severe trauma rather than treatment‐related complications. Although a subclavian steal phenomenon after TEVAR was frequent in ultrasound, the rate of symptomatic subclavian steal was low. Our data therefore align with guideline‐driven selective revascularization strategies when left subclavian coverage is required [3].

Limitations include the retrospective single‐center design, limited follow‐up (predominantly due to tourism‐related repatriation), and the inability to compare TEVAR with open repair.

Nonetheless, our observation underscores the need for multidisciplinary pathways that integrate vascular, trauma, radiology, neurology, and rehabilitation expertise for patients with BTAI. As BTAI patients are primarily managed on surgical services, systematic neurologic screening and early linkage to neurorehabilitation may represent actionable opportunities to improve long‐term independence.

## Author Contributions

**Lukas Mayer‐Suess:** conceptualization, data curation, formal analysis, investigation, methodology, validation, visualization, writing – original draft. **Maximilian Lutz:** conceptualization, data curation, formal analysis, investigation, methodology, validation, visualization, writing – original draft. **David Wippel:** conceptualization, data curation, formal analysis, investigation, methodology, validation, visualization, writing – original draft. **Kurt Moelgg:** data curation, investigation, validation, writing – review and editing. **Florian Frank:** data curation, investigation, validation, writing – review and editing. **Stefan Kiechl:** funding acquisition, methodology, project administration, resources, supervision, validation, writing – review and editing. **Sabine Wipper:** methodology, project administration, resources, supervision, validation, writing – review and editing. **Elke R. Gizewski:** methodology, project administration, resources, supervision, validation, writing – review and editing. **Michael Knoflach:** conceptualization, methodology, project administration, resources, supervision, validation, writing – review and editing. **Martin Freund:** methodology, project administration, supervision, validation, writing – review and editing. **Alexander Loizides:** conceptualization, methodology, project administration, resources, supervision, validation, writing – review and editing. **Florian Enzmann:** conceptualization, methodology, project administration, resources, supervision, validation, writing – review and editing. None assigned: software.

## Conflicts of Interest

The authors declare no conflicts of interest.

## Data Availability

The data that support the findings of this study are available from the corresponding author upon reasonable request.
